# Synaptotagmin Ca^2+^ Sensors and Their Spatial Coupling to Presynaptic Ca_v_ Channels in Central Cortical Synapses

**DOI:** 10.3389/fnmol.2018.00494

**Published:** 2019-01-15

**Authors:** Grit Bornschein, Hartmut Schmidt

**Affiliations:** Carl-Ludwig Institute for Physiology, Medical Faculty, University of Leipzig, Leipzig, Germany

**Keywords:** Synaptotagmin, release sensor, Ca^2+^ channel, coupling distance, nanodomain, microdomain

## Abstract

Ca^2+^ concentrations drop rapidly over a distance of a few tens of nanometers from an open voltage-gated Ca^2+^ channel (Ca_v_), thereby, generating a spatially steep and temporally short-lived Ca^2+^ gradient that triggers exocytosis of a neurotransmitter filled synaptic vesicle. These non-steady state conditions make the Ca^2+^-binding kinetics of the Ca^2+^ sensors for release and their spatial coupling to the Ca_v_s important parameters of synaptic efficacy. In the mammalian central nervous system, the main release sensors linking action potential mediated Ca^2+^ influx to synchronous release are Synaptotagmin (Syt) 1 and 2. We review here quantitative work focusing on the Ca^2+^ kinetics of Syt2-mediated release. At present similar quantitative detail is lacking for Syt1-mediated release. In addition to triggering release, Ca^2+^ remaining bound to Syt after the first of two successive high-frequency activations was found to be capable of facilitating release during the second activation. More recently, the Ca^2+^ sensor Syt7 was identified as additional facilitation sensor. We further review how several recent functional studies provided quantitative insights into the spatial topographical relationships between Syts and Ca_v_s and identified mechanisms regulating the sensor-to-channel coupling distances at presynaptic active zones. Most synapses analyzed in matured cortical structures were found to operate at tight, nanodomain coupling. For fast signaling synapses a developmental switch from loose, microdomain to tight, nanodomain coupling was found. The protein Septin5 has been known for some time as a developmentally down-regulated “inhibitor” of tight coupling, while Munc13-3 was found only recently to function as a developmentally up-regulated mediator of tight coupling. On the other hand, a highly plastic synapse was found to operate at loose coupling in the matured hippocampus. Together these findings suggest that the coupling topography and its regulation is a specificity of the type of synapse. However, to definitely draw such conclusion our knowledge of functional active zone topographies of different types of synapses in different areas of the mammalian brain is too incomplete.

## Introduction

The release of neurotransmitter from presynaptic terminals and its modulation via synaptic plasticity are the bedrocks of directed information flow within neuronal circuits of the central nervous system (CNS). An action potential (AP) triggers the release of a neurotransmitter filled synaptic vesicle (SV) by opening voltage-gated Ca^2+^ channels (Ca_v_s) in the presynaptic active zone (AZ). The inflowing Ca^2+^ diffuses toward the SV, which bears the primary Ca^2+^ sensor proteins Synaptotagmin (Syt) 1 or 2 on its surface that are required for triggering its fusion with the presynaptic plasma membrane. Ca^2+^ binding to Syt changes its conformation and the resulting interaction with proteins of the core release machinery, the soluble *N*-ethylmaleimide-sensitive factor attachment protein receptor (SNARE) proteins, and other proteins at the AZ ultimately triggers the fusion of the SV with the presynaptic plasma membrane (Südhof, 2013; Kaeser and Regehr, 2014). Thus, although aspects of this process are still not understood, it can be noted that Syts link Ca^2+^ influx to SNARE mediated SV fusion.

The process of transmitter release is probabilistic, i.e., not every AP leads to exocytosis; rather it triggers the release of a SV only with a certain probability. The average vesicular release probability (*p*_r_) can be quantified by way of analyzing fluctuations in postsynaptic current amplitudes (PSCs) under conditions of different *p*_r_, e.g., at different concentrations of extracellular Ca^2+^ ([Ca^2+^]_e_; Clements and Silver, 2000). Instead of recording PSCs, recently it became also feasible to more directly monitor glutamate release from individual boutons by imaging the fluorescence of a genetically encoded, intensity-based glutamate-sensing fluorescent reporter (iGlusnFr; Jensen et al., 2017; Helassa et al., 2018; Marvin et al., 2018).

The initial *p*_r_ (*p*_r1_) is an important factor not only in determining the release fidelity for a single AP but also in setting the properties of short-term plasticity of a synapse (Zucker and Regehr, 2002; Abbott and Regehr, 2004). This can be illustrated by a simple example of paired-pulse ratio (PPR) in the absence of replenishment of SVs between the two APs of a paired-pulse experiment. In this case PPR = *p*_r2_/*p*_r1_ (1-*p*_r1_), where *p*_r2_ is the release probability of the second release process, which may differ from *p*_r1_. In general, it can be noted that if *p*_r1_ > 0.5 the synapse will depress, i.e., PPR < 1, and only the magnitude of the depression will depend on *p*_r2_. However, if *p*_r1_ < 0.5 the synapse will show facilitation or depression depending on *p*_r2_.

The *p*_r_ depends on the Ca^2+^-binding kinetics of the release machinery, i.e., the Ca^2+^-binding kinetics of Syt in the context of the SNARE and other proteins at the AZ, and on the amplitude and time course of the Ca^2+^ signal “seen” by Syt. The latter depends on different factors, including the number and types of Ca_v_s, their diffusional distance to Syt, and the characteristics of other Ca^2+^-binding proteins present in the terminal. Ca^2+^ entering the presynaptic terminal builds a steep, short-lasting concentration gradient around the mouth of the open Ca_v_s that rapidly diminishes with increasing distance from the channel. Due to the steepness and short duration of this Ca^2+^ gradient a chemical equilibrium is never established in this process. This makes the intracellular Ca^2+^-binding kinetics of the release sensor, rather than its affinity alone, as well as its diffusional distance to the Ca_v_s crucial to the control of speed and reliability of transmitter release (Bollmann et al., 2000; Schneggenburger and Neher, 2000; Eggermann and Jonas, 2012). In this review we will focus on these two prominent factors in the regulation of *p*_r_, the synaptic Ca^2+^-binding kinetics of Syt proteins and their topographical relationships to Ca_v_s. We will put an emphasis to more recent findings at small synapses in cortical structures of the mammalian brain.

## Properties of Release Sensors for Synchronous Release

Synaptotagmin–1,−2, and −9 (Syt1, 2, 9) are the known Ca^2+^ sensors for fast, synchronous transmitter release in the millisecond time window following an AP (Südhof, 2014). Syt1 and Syt2 are the dominating Syt isoforms for synchronous release in the mammalian brain while Syt9 expression appears to be restricted to the limbic system and the striatum (Berton et al., 1997; Fox and Sanes, 2007; Xu et al., 2007). In addition to fast synchronous release, a second, slow and asynchronous component of transmitter release has been described (Geppert et al., 1994; Goda and Stevens, 1994). Asynchronous release is primarily activated during and following repetitive stimulation and operates via sensors different from those for synchronous release (Sun et al., 2007; Kochubey et al., 2011). Due to their dominating role for rapid neuronal communication, we will focus here on Syt1 and Syt2 triggered release processes. Molecular and structural aspects of Syt1, 2 proteins and their interactions with SNARE- and scaffold proteins were covered by several comprehensive recent reviews (Südhof and Rothman, 2009; Südhof, 2012, 2013; Kaeser and Regehr, 2014; Brunger et al., 2018; Park and Ryu, 2018).

Briefly, a synaptic vesicle bears approximately 15 copies of Syt on its surface (Takamori et al., 2006). Each Syt has two C2 domains that constitute Ca^2+^-binding and in addition might mediate protein-protein interactions with SNAREs and other Syt proteins or interactions with the membrane. One of the C2 domains is a C2A domain that binds three Ca^2+^ ions, while the other one is a C2B domain that binds two Ca^2+^ ions (Südhof, 2013). Upon Ca^2+^ binding Syt triggers rapid synchronous vesicle fusion but the detailed molecular mechanisms are complex and still controversial (Brunger et al., 2018; Park and Ryu, 2018). Some of the proposed models discuss the role of Syt in at least two processes: First, prior to Ca^2+^ influx spontaneous fusion of synaptic vesicles has to be prevented by inhibiting the constititively active SNARE complex from full zippering (SNARE clamping). Second, upon Ca^2+^ influx fusion is triggered by relieving SNAREs from the clamp (SNARE unclamping). Among the proposed models it is under debate if SNARE clamping is mediated directly by Syt or if and in as much it involves a second protein called Complexin (Cpx), which is discussed to also have a SNARE clamping function (Südhof, 2013; Trimbuch and Rosenmund, 2016), and can form a protein complex with SNAREs and Syt (Zhou et al., 2017). Hence, according to these models, Syt either has a dual function by first clamping SNARE zippering and an uncalmping function by relieving the clamp upon Ca^2+^ binding or only by relieving a Cpx-mediated SNARE clamp upon Ca^2+^ binding. Other models emphasize the membrane binding properties of Syt and suggest that membrane insertion of Ca^2+^-bound Syt could cross-link vesicle and plasma membrane or lower the energy barrier for fusion by either regulating the vesicle to plasma membrane distance or by locally curving the plasma membrane. In addition, there is evidence that Syt also directly binds to Ca^2+^ channels (Sheng et al., 1997). Since a detailed discussion of the molecular mechanisms of the fusion process is beyond the scope of this review, we refer the reader to most recent reviews (Trimbuch and Rosenmund, 2016; Brunger et al., 2018; Park and Ryu, 2018). We will focus here on the kinetic aspects of the interaction between Ca^2+^ ions and Syt1, 2.

### Synaptic Ca^2+^-Binding Kinetics of Synaptotagmins

**Figure 1:**
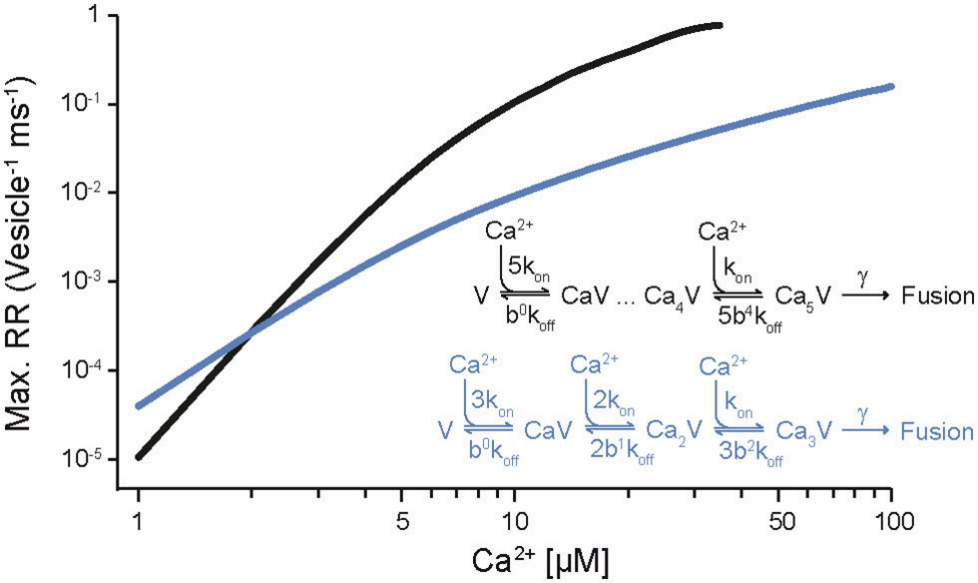
Dependency of release rates on the intracellular Ca^2+^ concentration. Release rates were calculated for different [Ca^2+^]_i_ using the sensor models developed for Syt2 at the calyx of Held (black; Schneggenburger and Neher, 2000) and for Syt1 at chromaffin cells (blue; Voets, 2000). Peak release rates per vesicle were plotted against the corresponding [Ca^2+^]_i_. Insets show the kinetic binding schemes for the reaction between vesicular sensor (V) and Ca^2+^.

It has been known for half a century that transmitter release has a non-linear, approximately power of 4 dependency on [Ca^2+^]_e_ (Dodge and Rahamimoff, 1967). However, a quantification of the intracellular presynaptic Ca^2+^-binding kinetics of a CNS release process became available only more recently (Bollmann et al., 2000; Schneggenburger and Neher, 2000), resulting in a detailed kinetic model of Ca^2+^-binding and release for the young calyx of Held synapse in the auditory brainstem (Figure 1), which expresses the Syt2 isoform as prime release sensor (Kochubey et al., 2016). The model was established based on an elegant combination of presynaptic Ca^2+^ uncaging and Ca^2+^ imaging with pre- and postsynaptic patch-clamp recordings (Box 1). The established model covers five cooperative, low-affinity Ca^2+^-binding sites with fast kinetic rate constants for Ca^2+^-binding and -unbinding (*k*_on_ ~10^8^ M^−1^s^−1^, *k*_off_ ~5000 s^−1^, respectively) and accounted for the experimental, cooperative power of 4 dependency of the release rate onto the intracellular Ca^2+^ concentration ([Ca^2+^]_i_) as well as brief synaptic delays (Figure 1, Table 1). It should be noted that this model does not reflect the Ca^2+^-binding kinetics of Syt2 alone but rather the kinetics of Syt2 embedded in its natural synaptic environment. For simplicity we will refer to it as the Syt2 model.

Box 1 **Quantification of the Ca^2+^-binding kinetics of Syt in presynaptic terminals via Ca^2+^ uncaging.**

Syt is integrated in the supra-molecular protein complex of the release machinery, which will influence its Ca^2+^-binding kinetics in a non-predictable manner similar to other Ca^2+^ sensor proteins (Xia and Storm, 2005). Consequently, Syt2 has been analyzed in Syt2 expressing synapses (cf. above). Toward this end, it was required to first define the local [Ca^2+^]_i_ at the release sensor and second, to quantify corresponding release rates.

At present it is difficult or even impossible to directly quantify the local [Ca^2+^]_i_ at the release sensor. Even if it were possible to measure [Ca^2+^]_i_ at areas as small as ~0.5 μm^2^ as performed at squid giant synapses (Llinás et al., 1992), the local [Ca^2+^]_i_ at the sensor would remain rather ill-defined due to the steep spatial gradient of synaptic [Ca^2+^]_i_ elevations, the unknown distance to the sensor and uncertainties about endogenous Ca^2+^ buffers (Neher, 1998a; Bucurenciu et al., 2008, 2010; Bornschein et al., 2013; Schmidt et al., 2013).

Ca^2+^ uncaging has been shown to be a useful method to resolve this problem (Heidelberger et al., 1994). Ca^2+^ uncaging elevates [Ca^2+^]_i_ uniformly in a presynaptic terminal. Due to this uniform [Ca^2+^]_i_ elevation throughout the terminal, local [Ca^2+^]_i_ will be identical to global [Ca^2+^]_i_, which in turn is quantified by concomitant Ca^2+^ imaging. Uniform elevations of synaptic [Ca^2+^]_i_ to different levels by flash photolysis of caged Ca^2+^ have been employed for establishing the relationship between [Ca^2+^]_i_ and release and permitted the construction of the above described Syt2-based release models. This method was applied at the giant calyx of Held (Bollmann et al., 2000; Schneggenburger and Neher, 2000; Lou et al., 2005; Sun et al., 2007; Kochubey and Schneggenburger, 2011), which permits direct whole-cell patch-clamp equilibration with caged Ca^2+^ compounds and Ca^2+^ indicator dyes, Ca^2+^ uncaging at the presynaptic terminal and concomitant patch-clamp recordings from the postsynaptic site. Thus, differences in PSC amplitudes and synaptic delays recorded at the postsynaptic site can be directly correlated to differences in [Ca^2+^]_i_ at the presynaptic release sensor. Finally, recording of quantal PSCs (“minis”) allows for calculating the release rates by deconvolution analysis (Van der Kloot, 1988; Diamond and Jahr, 1995; Bollmann et al., 2000; Schneggenburger and Neher, 2000; Neher and Sakaba, 2001). Deconvolution decomposes the PSC into the times of release of individual quanta, thereby, giving the release rate in quanta/s during the PSC. The recorded mini serves as elementary quantal event for the deconvolution. Deconvolution assumes that there are no quantal interactions at the synapse, i.e., the PSC arises from linearly summing minis.

**Table 1:** Parameters of release sensors.

**Model No. /Parameter**	**1**	**2**	**3**	**4**	**5 young**	**5 mature**	**6**	**Unit**
k_on_	1	0.9	0.9	3	1.21	1.15	0.044	×10^−8^ M^−1^s^−1^
k_off_	4000	9500	3000	3000	6500	7900	56	s^−1^
*b*, cooperativity factor	0.5	0.25	0.25	–	0.26	0.26	1	
*I*_+_, basal fusion rate	2	–	–	–	–	–	–	×10^−4^ s^−1^
*f*, increase upon Ca^2+^ binding	31.3	–	–	–	–	–	–	
γ, release rate	-	6000	5000	40000	6960	6960	1450	s^−1^
*γ2*, forward isomerization	–	–	–	30000	–	–	–	s^−1^
δ, backward isomerization	–	–	–	8000	–	–	–	s^−1^
k_priming_	–	–	0.05	–	–	–	–	×10^−8^ M^−1^s^−1^
k_unpriming_	–	–	50	–	–	–	–	s^−1^
k_filling_	–	–	8	–	–	–	–	s^−1^
k_unfilling_	–	–	12	–	–	–	–	s^−1^
k_basal_	–	–	2	–	–	–	–	s^−1^

**Scheme 1:**
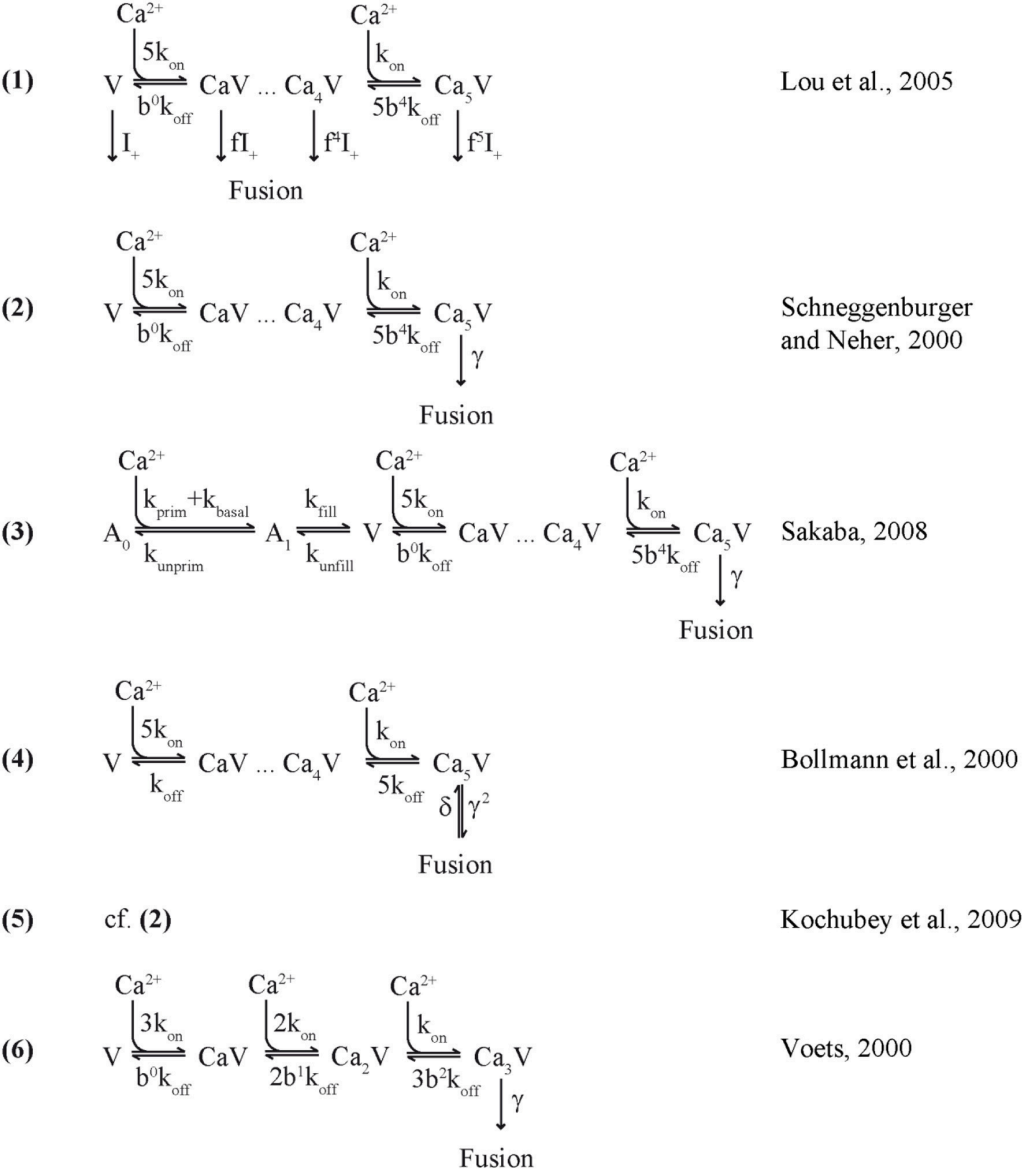
Release sensor models.

In following work the Syt2 model has been extended (Scheme 1) to also account for release at low [Ca^2+^]_i_ (Lou et al., 2005), for phasic and tonic release (Millar et al., 2005; Pan and Zucker, 2009), for asynchronous release (Sun et al., 2007), and to address mechanisms of synaptic plasticity (Felmy et al., 2003; Sakaba, 2008; Pan and Zucker, 2009; Bornschein et al., 2013; Brachtendorf et al., 2015). In addition, it has been shown that the intracellular Ca^2+^ sensitivity of Syt2-driven release is slightly reduced between postnatal day (P) 8-9 and P12-15 at the calyx of Held (Wang et al., 2008; Kochubey et al., 2009). Currently, the established Syt2 models are widely used in quantitative descriptions of transmitter release (Eggermann et al., 2012; Stanley, 2016).

For mammalian CNS synapses, the Syt2-based models were originally constructed at the young (1–2 weeks old) calyx of Held but subsequently also at a small CNS synapse, the immature cerebellar basket cell to Purkinje cell (PC) synapse (Sakaba, 2008), at which Syt2 also represents the dominant Syt isoform (Chen et al., 2017). Notably, Syt2 is the dominating fast release sensor in hindbrain structures, while in most forebrain structures, including the neocortex, Syt1 is the sensor mediating fast synchronous release (Berton et al., 1997; Fox and Sanes, 2007; Xu et al., 2007). Importantly, a detailed kinetic model similar to that of Syt2 is at present not available for Ca^2+^-binding to Syt1 in mammalian CNS synapses. At very young, P5-6 pre-calyx synapses in the brainstem a fast release component has been reported to be mediated via Syt1 but no kinetic model has been constructed (Kochubey et al., 2016). This Syt1 triggered release process had a less than power of 2 dependency on [Ca^2+^]_i_, i.e., its [Ca^2+^]_i_ dependency was substantially shallower than that of Syt2 triggered release at the young calyx of Held. A kinetic model for Syt1-mediated release has been constructed for fusion of dense core vesicles at chromaffin cells of the adrenal gland (Voets, 2000; Sørensen et al., 2003). In this model three to four Ca^2+^-binding sites with rate constants of about two orders of magnitude smaller than those for the synaptic Syt2 model were found to be suitable to describe secretion from chromaffin cells, which is much slower than rapid synaptic release (Table 1). Consequently, the resulting dependency of the release rate onto [Ca^2+^]_i_ was again much shallower than for synaptic Syt2 (Figure 1). Also, a quantitative comparison of the dependency on [Ca^2+^]_e_ of release processes mediated by Sy1 and Syt2 in cultured neurons revealed differences between the two proteins. Finally, differences were found for the kinetics of Syt1 and Syt2 mediated postsynaptic currents (PSCs), indicating differences in the kinetics of Syt1 vs. Syt2 triggered release. Consequently, it has been suggested that the expression of a particular Syt isoform dictates the properties of release at its synapse (Xu et al., 2007). Thus, it will be interesting to see, whether Syt1-triggered release at mature synapses in the mammalian brain indeed has a dependency on [Ca^2+^]_i_ different from Syt2-triggered release.

### Kinetics of Ca^2+^-Unbinding From Syt, Active Ca^2+^, and Facilitation Sensors

Paired pulse facilitation (PPF) is a form of short-term synaptic plasticity important for synaptic computation (Abbott and Regehr, 2004). PPF is a use-dependent enhancement of transmitter release following the second of two successive APs separated by a millisecond time interval. Although PPF was discovered more than 70 years ago, its mechanisms remain controversial and may differ between synapses (Zucker and Regehr, 2002). Originally, it has been suggested that “Ca^2+^ remaining attached to specific sites on the inner axon membrane” causes facilitation. For this Ca^2+^ the term “active Ca^2+^” was coined (Katz and Miledi, 1968). In a simpler form of the “residual Ca^2+^ hypothesis” a residue of free Ca^2+^ ([Ca^2+^]_res_) from the first AP summates with free Ca^2+^ ([Ca^2+^]_i_) from the second AP, thereby, causing amplified release. However, it has been recognized early that the decay of [Ca^2+^]_res_ deviates from the time course of facilitation, such that [Ca^2+^]_res_ cannot fully account for facilitation (Blundon et al., 1993). Additionally, due to the large amplitude difference between [Ca^2+^]_res_ (~100 nM) and nano- or microdomain [Ca^2+^]_i_ at the release site during the second AP (~20–100 μM) simple Ca^2+^ summation is unlikely to be the exclusive source of facilitation (Zucker and Regehr, 2002). Consequently, at different synapses different conceptions were developed to account for facilitation. These include slow Ca^2+^ relaxation of the bound sensor (Yamada and Zucker, 1992; Bertram et al., 1996; Matveev et al., 2002), separate sites for release and facilitation (Atluri and Regehr, 1996), elevated release site [Ca^2+^]_i_ during the second pulse (Geiger and Jonas, 2000; Felmy et al., 2003; Bollmann and Sakmann, 2005), buffer effects (Neher, 1998a; Rozov et al., 2001), or activity dependent recruitment of additional release sites (Valera et al., 2012; Miki et al., 2016; Doussau et al., 2017). For a recent comprehensive review on mechanisms of PPF (see Jackman and Regehr, 2017).

Here, we focus on Syt-related mechanisms of PPF. Ca^2+^-unbinding from the release sensor has been suggested as one mechanism of PPF (Yamada and Zucker, 1992; Bertram et al., 1996; Matveev et al., 2002). Young cerebellar PCs are connected among each other via recurrent GABAergic synapses that show PPF during high-frequency activation. Although PCs strongly express the “slow” and “fast” native Ca^2+^ buffers Parvalbumin (PV) and Calbindin-D28k (CB), respectively, PPF was not affected by loss of either of the buffers (Bornschein et al., 2013). Rather the results indicated that a residue of Ca^2+^ remaining bound to the release sensor Syt2 (Schneggenburger and Neher, 2000; Sakaba, 2008) after the first AP is the probable main cause of PPF at PC to PC synapses, a mechanism highly reminiscent of the original “active Ca^2+^” mechanism (Katz and Miledi, 1968).

Another suggested mechanism reminding on the original “active Ca^2+^” mechanism was that a facilitation sensor separate from the release sensor could exist (Atluri and Regehr, 1996). The molecular identity of the facilitation sensor, however, remained elusive until recently Syt7 has been identified to operate as a facilitation sensor (Jackman et al., 2016). Syt7 is abundantly found in presynaptic plasma membranes (Li et al., 2017), while Syt1 and Syt2 rather locate to SV membranes. The intrinsic Ca^2+^ affinities of Syt1 and Syt7 are comparably low in solution (*K*_D_ ≥ 100 μM; Radhakrishnan et al., 2009; Voleti et al., 2017). In the presence of lipids the apparent Ca^2+^ affinity of both proteins increases, albeit for Syt7 stronger than for Syt1, such that the apparent Ca^2+^ affinity of Syt7 is ~10fold higher than that of Syt1 (Sugita et al., 2002). The apparent Ca^2+^-sensing properties of Syt1 and Syt7 correlate with their specific functions, such that Syt1 is activated only by high Ca^2+^ concentrations (~10–100 μM) typical for AP-evoked [Ca^2+^]_i_ elevations in the vicinity of Ca^2+^ channels, while Syt7 can also operate during longer lasting increases in residual Ca^2+^ in the low micromolar range (Volynski and Krishnakumar, 2018). These characteristics made Syt7 a promising candidate for the proposed facilitation sensor. Indeed, it was found that Syt7 contributes to PPF at different types of facilitating synapses in the hippocampus and at cortico-thalamic synapses. Mechanistically, Ca^2+^-binding to the C2A domain of Syt7 was required for facilitation (Jackman et al., 2016; Jackman and Regehr, 2017; Turecek et al., 2017).

For Syt7 at least two other functions were reported (Volynski and Krishnakumar, 2018): It was found to act as a Ca^2+^ sensor for SV replenishment (Liu et al., 2014) and to mediate slow, asynchronous transmitter release (Bacaj et al., 2013; Luo and Südhof, 2017). Interestingly, the different proposed functions of Syt7 need not be mutually exclusive (Chen and Jonas, 2017). Consistently, it was found at cerebellar PF to PC synapses that Syt7 is involved in mediating both, PPF and asynchronous release (Turecek and Regehr, 2018). PPF at PF synapses has further been reported to depend on rapid SV replenishment with recruitment of additional release sites that resulted in an activity dependent, transient increase in the RRP (Valera et al., 2012; Brachtendorf et al., 2015; Miki et al., 2016; Doussau et al., 2017). It is tempting to speculate that the “overfilling” of an RRP by additional release sites could involve Syt7.

## Spatial Coupling Between Synaptotagmin and Ca_v_s

Besides the Ca^2+^-binding kinetics of Syt, its spatial relationship to the presynaptic Ca_v_s is crucial for setting fundamental synaptic properties, including *p*_r_, synchronicity of release and synaptic delays (Bucurenciu et al., 2008). The distance between Syt and the Ca_v_s is frequently referred to as the coupling distance. In general it may be asserted that for AP evoked release a tight coupling favors high *p*_r_ (Bucurenciu et al., 2008; Baur et al., 2015; Kusch et al., 2018), short synaptic delays (Bucurenciu et al., 2008), energy efficacy (Eggermann et al., 2012; Lu et al., 2016) and renders the release process less modifiable by Ca^2+^ buffers (Adler et al., 1991; Eggermann and Jonas, 2012; Bornschein et al., 2013; Schmidt et al., 2013; Brachtendorf et al., 2015). Loose coupling, on the other hand, offers more options for regulation and plasticity (Nadkarni et al., 2012; Vyleta and Jonas, 2014). There has been a comprehensive review on influx-release coupling at mammalian synapses of the peripheral NS (PNS) and the CNS (Eggermann et al., 2012). However, since then, a large body of work at AZs focused directly or indirectly on coupling distances and greatly advanced our knowledge about coupling and its regulation at different synapses. Some of these insights stem from classical model synapses, like the calyx of Held in the auditory brainstem, the frog neuromuscular junction, the squid giant synapse, or chick ciliary ganglion cells, which offer favorable conditions for electrophysiological analysis, in particular due to their large size. Insights from these synapses were covered by two recent comprehensive reviews (Wang and Augustine, 2015; Stanley, 2016) to which we refer the reader here. We will review advances in understanding AP-mediated Ca^2+^ influx-evoked transmitter release coupling at mammalian cortical AZs as well as their regulation during postnatal development and emerging roles of specific proteins in this regulatory process.

### Coupling Topographies

We will start this chapter with a brief note on nomenclature. The border between “tight” and “loose” coupling is not clearly defined (Box 2). A border line in the range of 70–100 nm has been suggested previously to distinguish between the two coupling regimes (Eggermann et al., 2012; Vyleta and Jonas, 2014). In light of the most recent quantitative estimates of coupling distances and domain topographies at mammalian CNS synapses (Table 2), we suggest an even narrower line of demarcation of no larger than 50 nm. At this coupling distance a given open Ca_v_ will be essentially ineffective in triggering release of a SV (Figure 3). Throughout this review we will use “loose coupling” if the coupling distance is ≥ 50 nm and “tight coupling” otherwise. In addition, we will use “single domain topography” (SDT) if only a single open Ca_v_ triggers release, and “domain overlap topography” (DOT) if a cluster of open Ca_v_s with overlapping Ca^2+^ signaling domains controls release. Finally, we will use “nanodomain” as synonym for tight coupling plus SDT and “microdomain” as synonym for loose coupling plus DOT (Fedchyshyn and Wang, 2005; Table 3; Box 3).

Box 2 **Deriving quantitative estimates of coupling distances.**

**Figure 2:**
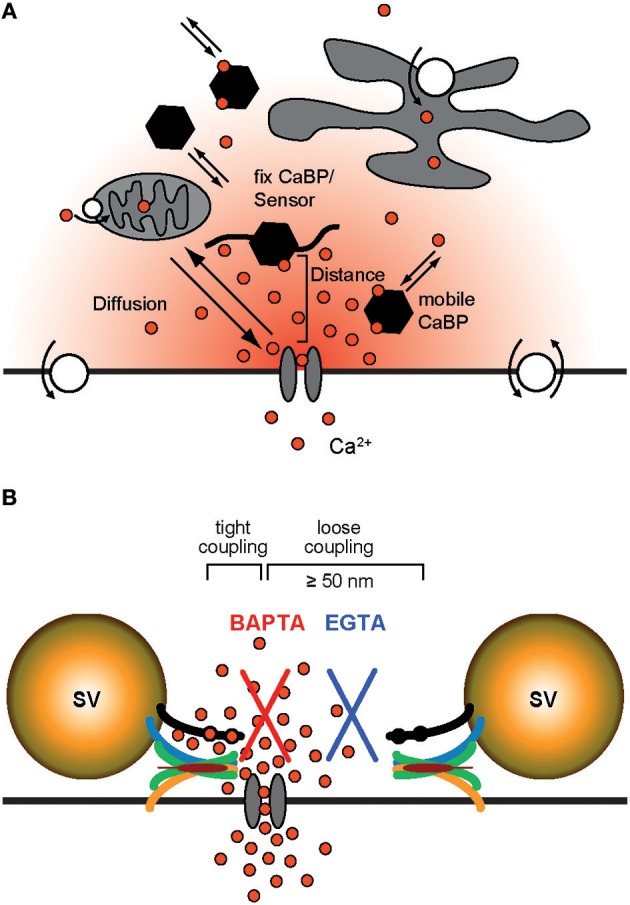
Ca^2+^ signaling domains. **(A)** General Ca^2+^ dynamics: Ca^2+^ enters a presynaptic terminal through a voltage-gated Ca^2+^ channel. Due to rapid diffusion (indicated by red gradient and the arrows) Ca^2+^ forms a steep, short-lived spatio-temporal gradient around the mouth of the open channel. It binds to mobile or fix Ca^2+^-binding proteins (CaBPs); some CaBP are pure buffers, others have an additional Ca^2+^ sensor function. Ultimately Ca^2+^ is cleared from the cytosol via Ca^2+^-ATPases (white circles with arrows) that either pump Ca^2+^ into the extracellular space or sequester it into organelles. **(B)** In a tight coupling regime a Syt bearing SV is located very close to the site of Ca^2+^ entry (<50 nm). If coupling is tight, at moderate concentrations only a buffer with rapid Ca^2+^ binding kinetics like BAPTA (red) can interfere with Ca^2+^ binding to Syt and prevent release. In a loose coupling regime, on the other hand, the SV is further away from the site of Ca^2+^ entry and also a slow buffer like EGTA (blue) can bind Ca^2+^ before it reaches the release sensor.

**Figure 3:**
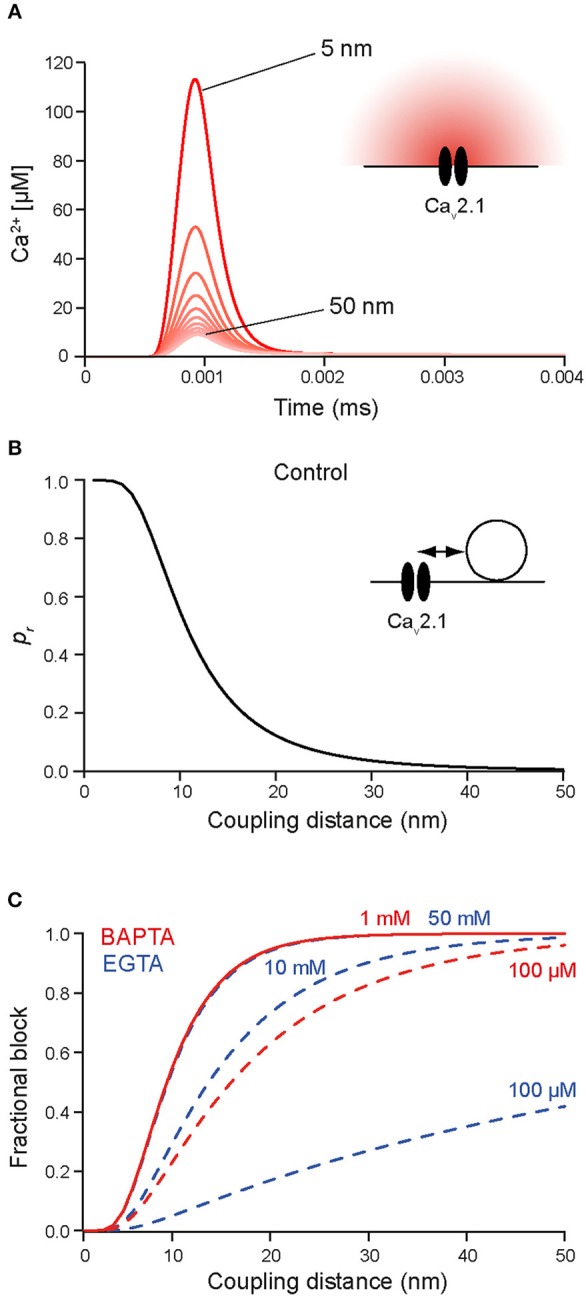
**(A)** Simulated [Ca^2+^]_i_ transients at increasing distances between 5 and 50 nm (5 nm increments) from a single Ca_v_2.1 channel (inset; Li et al., 2007) opening during an AP in the absence of Ca^2+^ buffers. **(B)** Release rates were simulated at increasing distances from the Ca_v_2.1 channel (1 nm increments) using the Syt2 sensor model from Figure 1. Release probabilities were calculated by integrating the release rates over time and plotted against the corresponding sensor-to-Ca_v_ coupling distances. Note the steep decline in *p*_r_ between 6 and 20 nm coupling distance and that *p*_r_ at 50 nm is almost 0. **(C)** Simulated relative reduction of *p*_r_ for different concentrations of EGTA (blue) and BAPTA (red). Moderate concentrations of EGTA are not very effective in blocking release close to a channel, while moderate concentrations of BAPTA are highly effective already at coupling distances of 10–20 nm. Higher concentration of EGTA mimic the effects of lower concentrations of BAPTA (concentrations are indicated). Note that in native boutons the concentrations of EGTA and BAPTA that yield corresponding effects on *p*_r_ will be different due to the presence of native Ca^2+^-binding proteins, which were not included in the simulations.

We are not aware of any report of a direct quantification of the coupling distance between Syt and Ca_v_s at AZs by microscopic techniques. In particular this appears to be due to the non-availability of appropriately sized labels. Hence, information about the average coupling distance is classically obtained by dialyzing a presynaptic terminus with exogenous Ca^2+^ chelators of similar affinity (*K*_D_) but different Ca^2+^-binding kinetics, i.e., different on-rates (kon; Adler et al., 1991; Augustine et al., 1991; Neher, 1998b; Eggermann et al., 2012). Typically the Ca^2+^ chelators EGTA (ethylene glycol-bis(2-aminoethylether)-N,N,N',N'-tetraacetic acid; *K*_D_ = 70 nM, *k*_on_ = 10^7^ M^−1^s^−1^; Nägerl et al., 2000) and BAPTA (1,2-bis(2-aminophenoxy)ethane-N,N,N',N'-tetraacetic acid; *K*_D_ = 220 nM, *k*_on_ = 4∗10^8^ M^−1^s^−1^; Naraghi and Neher, 1997) are used for this approach since they have similar *K*_D_ values but BAPTA is ~40 times faster than EGTA. Ca^2+^ chelators suppress synaptic transmission by reducing the amount of Ca^2+^ that binds to Syt (Figure 2). The exact amount of interference depends on four factors: the average coupling distance, the mobility of the chelator, its *k*_on_, and its concentration. If influx-release coupling is tight, only a chelator with a rapid *k*_on_ like BAPTA is able to capture Ca^2+^ in the nanodomain in the immediate vicinity of the channel before it reaches Syt, while chelators with slow *k*_on_ like EGTA fail to influence the nanodomain Ca^2+^. Thus, at moderate concentrations only the fast BAPTA will reduce the amount of transmitter released in a tight coupling regime. In a loose coupling regime, on the other hand, both, BAPTA and EGTA will interfere with transmitter release since Ca^2+^ has to diffuse a larger distance from the Ca_v_s to reach the sensor. This allows also the slower EGTA to capture Ca^2+^ in the microdomain before the ions reach the release sensor. Using this exogenous chelator dialysis approach, most of the estimates of coupling distances reviewed here were derived. It should be noted that the degree of interference actually not only depends on the *k*_on_ but also on the concentration of the buffer, i.e., a large concentration of EGTA interferes with release similar to a much smaller concentration of BAPTA (Figure 3). In order to obtain quantitative values of the coupling distance, additional information about the magnitude and duration of the Ca^2+^ influx and potential Ca^2+^ sensor saturation is required (e.g., Bucurenciu et al., 2008; Schmidt et al., 2013; Nakamura et al., 2015, 2018; Kusch et al., 2018). Finally, by combining all results in experimentally constrained computer simulations quantitative estimates of the average coupling distance can be obtained (Bucurenciu et al., 2008; Bornschein et al., 2013; Schmidt et al., 2013; Vyleta and Jonas, 2014; Nakamura et al., 2015; Kusch et al., 2018).

The exogenous chelator dialysis approach was applied to large synapses that can be directly infused with chelator containing solution (Adler et al., 1991; Borst and Sakmann, 1996) and to large neurons that permit dialyzing the distant presynaptic sites by prolonged somatic whole-cell patch-clamp recordings (Ohana and Sakmann, 1998; Bucurenciu et al., 2008; Bornschein et al., 2013). The advantage of this approach is that the intracellular concentrations of the Ca^2+^ chelators are well-defined.

Another way of loading neurons with exogenous chelators is by application of membrane permeant acetoxymethyl ester variants of the Ca^2+^ chelators (EGTA-AM or BAPTA-AM) to the extracellular bath solution (Atluri and Regehr, 1996; Matsui and Jahr, 2003; Hefft and Jonas, 2005). The AM-chelator compound passes the lipophilic plasma membrane and enters the presynaptic cytosol. There, the ester group is cleaved by enzymes, which makes the chelator membrane-impermeable. Depending on loading time, its intracellular concentration can substantially exceed its bath concentration due to continuous intracellular accumulation of the chelator as long as its AM-form is present in the bath. The advantage of this approach is its relative experimental ease and that it is well-tolerated also by small neurons. It has the disadvantage that the intracellular chelator concentration remains rather ill defined. Thus, it permits a rapid initial assessment of relative differences in coupling e.g., between age groups, if differently aged synapses are compared under otherwise identical experimental conditions (Matsui and Jahr, 2003; Hefft and Jonas, 2005; Baur et al., 2015).

Neurons express endogenous Ca^2+^ buffers with quantified Ca^2+^-binding kinetics (Lee et al., 2000; Faas et al., 2007). Knowledge about the expression of specific native Ca^2+^ buffers and there Ca^2+^-binding kinetics offers an alternative route to deriving quantitative estimates of coupling distances by comparing transmitter-release from wild-type terminals to release from mutant terminals lacking a specific native buffer (Bornschein et al., 2013; Schmidt et al., 2013).

**Table 2:** Quantitative estimates of coupling topographies at mammalian CNS synapses.

**Synapse, age**	**Brain region, preparation, species**	Average coupling distance (nm)^*^	**No of Ca_**v**_s controlling a release site, Ca_**v**_ subtypes**	**References**
BC – GC, P18-21	Hippocampus, Slice, Rat	10–20	≤3, Ca_v_2.1 (STD – DOT)	Bucurenciu et al., 2008, 2010
MF – CA3 PN, P20-23	Hippocampus, Slice, Rat	~75	n.d.	Vyleta and Jonas, 2014
CA3 – CA1 PN	Hippocampus, *in silico*	300	~70 (DOT)	Nadkarni et al., 2012
CA3 – CA1 PN, P14-21	Hippocampus, Slice, Mouse	≤30	1, Ca_v_2.1, Ca_v_ 2.2, (SDT)	Scimemi and Diamond, 2012
Hippocampal synapses	Hippocampus, Cell culture	25–70	2–14, Ca_v_2.1, Ca_v_ 2.2, Ca_v_ 2.3 (DOT)	Ermolyuk et al., 2013
PC – PC, P7-12	Cerebellum, Slice, Mouse	20–35	n.d.	Bornschein et al., 2013
PF – PC, P21-21	Cerebellum, Slice, Mouse	10–24	1, Ca_v_2.1 (SDT)	Schmidt et al., 2013; Baur et al., 2015; Kusch et al., 2018
PF – PC,P8-10	Cerebellum, Slice, Mouse	~60	≥3, Ca_v_2.1, Ca_v_2.2 (DOT)	Baur et al., 2015; Kusch et al., 2018
BC – PC, P14-16	Cerebellum, Slice, Mouse,	10–20	n.d., Ca_v_2.1	Arai and Jonas, 2014
MF – GC, P21-61	Cerebellum, Slice, Mouse	~7–20	Ca_v_2.1 (SDT)	Ritzau-Jost et al., 2014, 2018; Delvendahl et al., 2015
CH, P8-12	Brainstem, Slice, Mouse	~23	Ca_v_2.1, Ca_v_2.2 (DOT)	Fedchyshyn and Wang, 2005; Wang et al., 2009
CH, P16-18	Brainstem, Slice, Mouse	~63	Ca_v_2.1 (SDT)	Fedchyshyn and Wang, 2005; Wang et al., 2009
CH, P7	Brainstem, Slice, Rat	~20	~29, Ca_v_2.1 (DOT)	Nakamura et al., 2015
CH, P14 (21)	Brainstem, Slice, Rat	~30	~26, Ca_v_2.1 (DOT)	Nakamura et al., 2015

* *Coupling distances need not be homogeneous (Scimemi and Diamond, 2012; Ermolyuk et al., 2013; Ritzau-Jost et al., 2018)*.

*BC, basket cell; CH, calyx of Held; GC, granule cell; MF, mossy fiber; P, postnatal day; PN, pyramidal neuron; PC, Purkinje cell; PF, parallel fiber; n.d., not determined*.

**Table 3:** Active zone topographies.

**AZ topgraphy**		**Release**	**Chelators**	**Submaximal Cd^**2+**^ block**	**Full subtype block**	**References**
**SINGLE-DOMAIN TOPOGRAPHY (NO DOMAIN-OVERLAP)**						
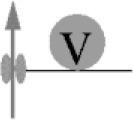	homog. tight coupling all *p_*r*_* identical = nanodomain	*p*_*r, avg*_ = 1/*N*∑*p*_*r*_ = *p*_*r*_ $F_{syn}={\left(1-p_{r}\right)}^{N}$ $P_{syn}=1-F=1-{\left(1-p_{r}\right)}^{N}$	BAPTA >> EGTA DE: Monophasic	PPR→	*RR*_*total*_ = *RR*_*P*/*Q*_ + *RR*_*N*_	Bucurenciu et al., 2008; Baur et al., 2015; Kusch et al., 2018
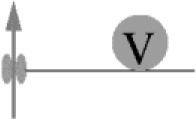	homog. loose coupling all *p_*r*_* identical	Ditto	BAPTA ≥ EGTA DE: Monophasic	Ditto	Ditto	Only simulation
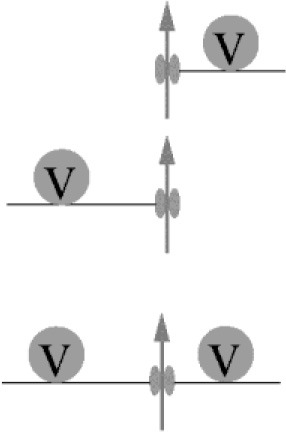	heterog. coupling heterog. *p_*r*_*	*p*_*r, avg*_ = 1/(*N*_1_ + *N*_2_)(∑*p*_*r*, 1_ + ∑*p*_*r*, 2_) $F_{syn}={\left(1-p_{r,1}\right)}^{N1}{\left(1-p_{r,2}\right)}^{N2}$ *P*_*syn*_ = 1−*F*	BAPTA ≥ EGTA DE: Biphasic	Ditto	Ditto	Scimemi and Diamond, 2012; Ritzau-Jost et al., 2018
**DOMAIN-OVERLAP TOPOGRAPHY**						
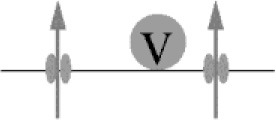	heterog coupling homog. *p_*r*_*	*Ca*_*total*_ = *Ca*_1_ + *Ca*_2_ *Ca*_*total*_ → *p*_*r*_	BAPTA ≥ EGTA DE: Monophasic	PPR↗	*RR*_*total*_ < *RR*_*P*/*Q*_ + *RR*_*N*_	Ermolyuk et al., 2013
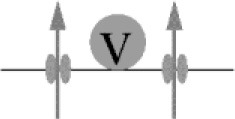	homog. tight coupling *homog.p_*r*_*	*Ca*_*total*_ = *nCa* *Ca*_*total*_ → *p*_*r*_	BAPTA >> EGTA DE: Monophasic	Ditto	Ditto	Nakamura et al., 2015
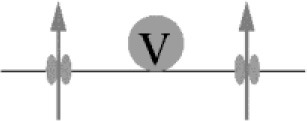	homog. loose coupling *homog. p_*r*_* = microdomain	Ditto	BAPTA ≥ EGTA DE: Monophasic	Ditto	Ditto	Vyleta and Jonas, 2014; Baur et al., 2015; Nakamura et al., 2015; Kusch et al., 2018

*p_r_, release probability of a vesicle; N, number of vesicles (or release sites that can release a max. of one vesicle), p_r,avg_, average release probabilities across vesicles; P_syn_, synaptic release probability, F_syn_, synaptic failure rate; DE, EGTA dose effect curve; PRR, paired-pulse ratio; RR, release rate*.

Box 3 **Estimating the functional domain topography.**

How many Ca_v_s open during an AP and do their Ca^2+^ signaling domains overlap? Immunolabelling techniques combined with electron microscopy provide highly valuable insights into the structural organization of Ca_v_ clusters and Ca_v_ subtypes at the AZ. Yet, in order to answer the above question for functional SDT or DOT they need to be combined with physiological studies at the synapse (Holderith et al., 2012; Baur et al., 2015; Nakamura et al., 2015; Kusch et al., 2018). Specifically, the use of the unspecific Ca_v_ blocker Cd^2+^ and/or a combination of Ca_v_ subtype specific blockers were shown to yield valuable insights into the functional domain topography (Table 3; Augustine et al., 1991; Mintz et al., 1995; Scimemi and Diamond, 2012).

At physiological temperature Cd^2+^ dissociates slowly from a Ca_v_ (Chow, 1991), thus, blocking a channel in an all-or-none fashion on the time-scale of an AP. The shape of Cd^2+^ dose-effect curves onto EPSC amplitudes as read-out for release will depend on the domain topography. If a presynaptic terminal harbors release sites with DOT, the curve will be non-linear whereas it will be linear if the release sites operate with SDT (Augustine et al., 1991). The construction of full dose-effect curves may be circumvented by analyzing the effects of a subsaturating concentration of Cd^2+^ onto the PPR (Scimemi and Diamond, 2012).

Application of a subsaturating concentration of Cd^2+^ reduces the amplitude of the first postsynaptic current (PSC) of a paired pulse experiment irrespective of the domain topography. However, its impact on the PPR markedly depends on whether the release sites operate with SDT or DOT. At a subsaturating concentration of Cd^2+^ some but not all Ca_v_s will be blocked during an AP. In a DOT, blocking some of the Ca_v_s controlling a synaptic vesicle will have effects similar to reducing [Ca^2+^]_e_, i.e., the initial *p*_r_ will be reduced while the PPR will increase. On the other hand, if a release site is controlled by a single Ca_v_ (SDT), release of synaptic vesicles encountering no Ca^2+^ would be blocked while release or facilitation of vesicles encountering Ca^2+^ would be the same as in the absence of Cd^2+^. In consequence, application of Cd^2+^ will increase PPR in a DOT but leave it unaltered in a SDT (Scimemi and Diamond, 2012).

These results can further be substantiated by using Ca_v_ subtype specific blockers, if more than one channel subtype contributes to release. For a SDT in which a given vesicle is linked to either of the Ca_v_ subtypes, the sum of the toxin-sensitive release fractions will not exceed release measured in the absence of toxin, i.e., the toxin sensitive release fractions will sum linearly. Contrariwise, for an AZ at which release of a vesicle is controlled in a DOT composed of different Ca_v_ subtypes, the sum of the blocked release fractions can exceed the control value (“supralinear” summation) because of the non-linear dependency of release on Ca^2+^ (Mintz et al., 1995; Scimemi and Diamond, 2012).

Tight influx-release coupling has been reported for giant synapses specialized for escape reflexes in the squid (Adler et al., 1991), bipolar cells in the goldfish retina (von Gersdorff and Matthews, 1994; Burrone et al., 2002) and for the frog neuromuscular junction (Harlow et al., 2001). The first descriptions of nanodomain coupling came from the squid giant synapse and chick ciliary ganglia cells (Adler et al., 1991; Stanley, 1993). In the mammalian CNS, inhibitory synapses in the hippocampus and cerebellum were found to operate at tight coupling and at least in part with SDT, i.e., with nanodomains (Bucurenciu et al., 2008, 2010; Eggermann and Jonas, 2012; Bornschein et al., 2013). Surprisingly, cortical glutamatergic synapses seemed to forgo the benefits of tight coupling. Experimental studies performed on young pyramidal neurons (PNs; Ohana and Sakmann, 1998; Rozov et al., 2001) and in hippocampal cell cultures (Ermolyuk et al., 2013) as well as computational models of hippocampal CA3 – CA1 PN synapses (Nadkarni et al., 2012) showed loose coupling and established the view that small glutamatergic synapses in the brain, in particular excitatory cortical synapses, operate at microdomain coupling (Eggermann et al., 2012).

Initial experimental evidence against the generality of this view was available from the CA3 – CA1 PN synapse (Scimemi and Diamond, 2012), showing SDT and results that were more consistent with a tighter coupling at this synapse rather than with DOT and a very large number of Ca_v_s loosely coupled to the release sensor as suggested by the above mentioned study of Nadkarni et al. (2012). In the cerebellar cortex of 3 to 4 weeks old mice, subsequent work quantified the coupling distance at the parallel fiber (PF) to PC synapse, an excitatory, glutamatergic synapse in the cerebellar cortex and probably the most abundant synapse in the mammalian brain. It was found that this synapse operates at tight coupling of ~24 nm (Schmidt et al., 2013). In successional work it was found that at this age coupling is not only tight but that these synapses operate with a nanodomain topography (Baur et al., 2015; Kusch et al., 2018) and that also further excitatory synapses in the cerebellar cortex operate with tight coupling (Ritzau-Jost et al., 2014; Delvendahl et al., 2015). Together these studies clearly contradicted the generality of microdomain coupling at excitatory synapses in mammalian cortical structures.

### Regulation of Coupling

An interesting difference between the experiments suggesting microdomain coupling in glutamatergic cortical synapses (Ohana and Sakmann, 1998; Rozov et al., 2001) and the experiments showing nanodomain coupling (Schmidt et al., 2013; Ritzau-Jost et al., 2014) was the age of the experimental animals: While the former studies were performed in young rats (~2 weeks old), in the latter studies, the coupling distance was assessed in more matured mice (>3 weeks old). Considering that the postnatal development of rats likely proceeds slower than that of mice, the age difference most likely corresponds to an even larger difference in brain maturation. This raised the possibility that the coupling distance could be regulated developmentally.

Support for the idea of a developmental regulation of coupling came from experiments performed at the calyx of Held. Experiments performed on young (~10 days old) and matured (~3 weeks old) calyces indicated a substantial developmental tightening of the coupling distance during postnatal development (Taschenberger et al., 2002; Fedchyshyn and Wang, 2005; Wang et al., 2008; Kochubey et al., 2009). Simulations quantified that the experimental results are explained by a developmental tightening of the spatial coupling between Ca^2+^ channels and Syt from ~60 to ~20 nm at the calyx of Held (Wang et al., 2009).

Coupling distances and domain topographies were quantified more recently in a developmental context (Table 2), again at the calyx of Held (Nakamura et al., 2015) and at the PF to PC synapse (Baur et al., 2015). At the calyx of Held, a moderate developmental tightening of the coupling distance between Syt and the closest Ca_v_ of a cluster from ~30 nm to ~20 nm between P7 and P14 was found, while the number of Ca_v_s within a cluster controlling a given release site remained relatively constant with an average in the range of 25 to 30 (Nakamura et al., 2015). Thus, although a developmental shortening of the coupling distance was found at the calyx of Held, it operated at fairly tight coupling with DOT, independent of age in the range of P7 to P14. By contrast, at the PF to PC synapse a switch from DOT to SDT was found. At ~P9, PF terminals operated with a DOT with a distance of ~60 nm between the closest Ca_v_ within a cluster and Syt, while at ~P23 a coupling distance of ~20 nm and SDT were found (Baur et al., 2015; Kusch et al., 2018). The DOT at young PF terminals was composed of Ca_v_2.1 (P/Q-type) and Ca_v_2.2 (N-type) concomitantly controlling a release site, with likely 2 Ca_v_2.1 and 1 Ca_v_2.2 triggering release during an AP. The nanodomain at more matured PF terminals comprised only a Ca_v_2.1 (Kusch et al., 2018).

These results may suggest that developmental tightening of the coupling distance is a common phenomenon in the mammalian brain, which could be accompanied by a switch from DOT to SDT in small, but not in large synapses. However, an alternative is that coupling distances, domain topographies and their regulation are synapse specific properties. At the glutamatergic MF to CA3 PN synapse a loose coupling distance of ~75 nm has been quantified in the matured hippocampus (Table 2; Vyleta and Jonas, 2014). However, this finding does not necessarily exclude a developmental tightening of the coupling distance at the MF to CA3 synapse. It remains possible that the young synapse operates at an even larger coupling distance. Thus, while developmental tightening and loose coupling in mature brain are not mutually exclusive, the finding of loose coupling at the mature MF – CA3 synapse clearly suggests that the coupling distance is a synapse specific property in the context of its developmental stage.

Are there other forms of regulation of the coupling distance besides developmental regulation? An intriguing possibility would be a regulation of the coupling distance depending on the activity of a synapse, i.e., as a mechanism of synaptic plasticity. Evidence for such use-dependent regulation of the coupling distance came from a recent study at hippocampal mossy fiber boutons (Midorikawa and Sakaba, 2017). It was found that increasing the level of cAMP in the boutons, which is a crucial step in the induction of long term potentiation, results in increased release from the bouton, while not increasing the number of synaptic vesicles in the RRP nor altering the Ca^2+^ influx. Based on the differential action of EGTA prior and following the induction of cAMP-mediated plasticity the study provides evidence for a tightening of the coupling distance following cAMP application (Midorikawa and Sakaba, 2017).

### Functional Considerations

The MF – CA3 PN synapse, which was found to operate at loose coupling in the matured hippocampus (Vyleta and Jonas, 2014), is highly plastic and expresses several forms of presynaptic plasticity (Salin et al., 1996). It has been suggested that loose coupling provides a molecular framework for high plasticity (Vyleta and Jonas, 2014). Consistent with this idea, synapses with tight coupling are mostly fast-signaling synapses in neuronal circuits specialized for high-frequency coding of sensory information or in motor control (Table 2). However, some of these synapses also show pronounced presynaptic plasticity. For example, the PF to PC synapse exhibits low-frequency depression and high-frequency facilitation (Doussau et al., 2017).

Loose coupling offers more possibilities for regulating transmitter release and plasticity, e.g., via the action of Ca^2+^ buffers, since in loose coupling also slow Ca^2+^ buffers can intercept sizable amounts of Ca^2+^ before it reaches Syt (Adler et al., 1991). In tight coupling regimes, on the other hand, only rapid buffers like BAPTA (Adler et al., 1991), Calretinin (Schmidt et al., 2013; Brachtendorf et al., 2015), or Calbindin (Bornschein et al., 2013) were found to be regulators of *p*_r_, while the “slow” buffer Parvalbumin (PV) did not affect *p*_r_ (Bornschein et al., 2013). At high concentrations, however, even PV becomes effective in affecting [Ca^2+^]_i_ and release in tight coupling regimes (Eggermann and Jonas, 2012). This is because PV actually is a rapid, high-affinity Ca^2+^ buffer but its Ca^2+^-binding sites also have a medium affinity for Mg^2+^ such that most binding sites are occupied by Mg^2+^ under physiological resting conditions and only a small amount of binding sites (~5%) are metal free (Lee et al., 2000). Thus, Ca^2+^-binding has to be preceded by Mg^2+^-unbinding, which proceeds with slow kinetics, i.e., the slow Mg^2+^-unbinding kinetics makes PV a slow Ca^2+^ buffer (Lee et al., 2000). However, if PV is expressed strongly in a synapse the small relative fraction of Mg^2+^-free binding sites can constitute a sufficiently large absolute concentration of rapidly Ca^2+^-binding PV to significantly affect [Ca^2+^]_i_ even in the nanodomain around a Ca_v_ channel. Metal free binding sites are then continuously replenished efficiently from the large pool of Mg^2+^-bound sites (Eggermann and Jonas, 2012). It should be noted that this action of PV is different from the effects of large concentrations of EGTA in tight coupling regimes. PV was already effective at concentrations ~500 μM due to rapid Ca^2+^-binding and replenishment via Mg^2+^-unbinding, while slow buffering by EGTA requires concentrations >10 mM to intercept [Ca^2+^]_i_ in the nanodomain.

Tight coupling increases speed and efficacy of synaptic transmission (Eggermann et al., 2012). In addition, it can provide an energy efficient design compared to loose coupling. To obtain a certain [Ca^2+^]_i_ level at the release sensor less Ca_v_s have to open in a tight than in a loose coupling regime (Eggermann et al., 2012). As the ATP cost of Ca^2+^ removal is a significant post of the presynaptic energy consumption (Kim et al., 2005), tight coupling can save energy. This, however, requires that Ca^2+^ influx would indeed be different between terminals with tight or loose coupling. Indeed results from the calyx of Held conform to this requirement, showing that concomitant with developmental coupling distance tightening the amplitudes of presynaptic Ca^2+^ transients decreased (Nakamura et al., 2015). On the other hand, at the PF – PC synapse presynaptic Ca^2+^ transients did not change developmentally despite the developmental switch from loose to tight coupling (Baur et al., 2015). Several Ca_v_s opening during the presynaptic AP no longer contributed to driving release at later developmental stages (Kusch et al., 2018). Their primary function remains speculative but could be in Ca^2+^-driven replenishment of synaptic vesicles into the readily releasable pool (Brachtendorf et al., 2015; Miki et al., 2016; Doussau et al., 2017).

### Molecular Regulators of the Coupling Distance

Ca^2+^ influx-transmitter release coupling is mediated via proteins of the AZ scaffold, albeit, this process is still not well-understood at present and a detailed review of the AZ scaffold is far beyond the scope of this paper. We briefly focus on some recent advances directly related to establishing influx—release coupling. RIMs (Rab3-interacting molecules) are known as central organizer of the AZ (Südhof, 2012). Specifically, they are required for recruiting Ca_v_2.1 and Ca_v_2.2 channels to the AZ (Kaeser et al., 2011), which can be considered as a first step in coupling Ca^2+^ influx to transmitter release, in particular since these channel subtypes are the most important ones for AP-mediated fusion (Table 2). The protein Septin5 was identified as a negative regulator of tight coupling during development, i.e., its downregulation was permissive to the establishment of tight coupling (Yang et al., 2010). Proteins involved in mediating tight coupling were identified more recently, suggesting RIM-BPs (RIM-binding proteins; Acuna et al., 2015; Grauel et al., 2016) and Munc13-3 (Ishiyama et al., 2014; Kusch et al., 2018), as positive regulators of the coupling distance. Thereby, Munc13-3 was found to be a developmental mediator of tight coupling (Kusch et al., 2018). However, details of the interplay between identified regulators of the coupling distance, their relationships to other regulatory proteins at the AZ, and details of their interaction with the exocytotic core complex remain essentially unclear.

## Concluding Remarks

More than 30 years after the steep non-linear dependency of transmitter release onto [Ca^2+^]_e_ has been established (Dodge and Rahamimoff, 1967), detailed kinetic five-site models of the [Ca^2+^]_i_ dependency of Syt2-triggered transmitter release were developed (Bollmann et al., 2000; Schneggenburger and Neher, 2000) and subsequently elaborated to cover sub-modes and subtleties of release (Lou et al., 2005; Sun et al., 2007; Pan and Zucker, 2009) and to capture developmental aspects (Kochubey et al., 2009). These models are widely applied in functional quantitative studies of transmitter release and AZ topography.

During the past decade several functional studies focused directly or indirectly on the coupling distance between Syts and Ca_v_s at mammalian cortical synapses. Initially it was thought that only GABAergic synapses in cortical structures make use of tight coupling, while cortical glutamatergic synapses seemed to operate with loose coupling (Ohana and Sakmann, 1998; Rozov et al., 2001; Eggermann et al., 2012; Nadkarni et al., 2012; Stanley, 2016). However, results from a glutamatergic synapse in the mature cerebellar cortex falsified the generality of this hypothesis (Schmidt et al., 2013). From subsequent work (Table 2) it became evident that synapses in the matured mammalian brain, including synapses in cortical structures of hippocampus and cerebellum, indeed make widespread use of tight coupling and, furthermore, that release of a SV was frequently triggered by opening of only a few or even a single Ca_v_. At synapses investigated in a developmental context, it was found that tight coupling at matured synapses develops from an initially loose coupling at younger synapses. This latter result provides an explanation why previous studies predominantly found microdomain coupling at glutamatergic cortical synapses. These earlier studies were performed at synapses of very young animals (Eggermann et al., 2012; Stanley, 2016). The concept of nanodomain coupling was developed 20 years ago at the squid giant synapse (Adler et al., 1991) and calyx-type synapses in the chick ciliary ganglion (Stanley, 1993) and now experiences a revival at matured mammalian central synapses.

However, tight coupling is not universal for synapses of the mature mammalian brain (Vyleta and Jonas, 2014). As suggested by Vyleta and Jonas, the present state of knowledge indicates that coupling distances are specific adaptations to the function of a synapse. GABAergic central synapses appear to operate at tight coupling, most probably irrespective of age (Table 2). For glutamatergic synapses the situation is more complex. While excitatory synapses specialized for rapid signaling develop a tight, nanodomain coupling topography, synapses highly adaptive via plasticity make use of loose coupling even in matured brain. To learn more about the rules that regulate coupling distances will require to investigate further types of synapses in different brain regions. For example, a particularly striking lack of quantitative data on coupling distances and AZ topographies exists for neocortical synapses (Eggermann et al., 2012; Stanley, 2016; Table 2). To our knowledge, a coupling distance has never been quantified at a neocortical synapse.

For understanding the rules regulating coupling, it will be also required to identify the proteins that link Syt bearing SVs to Ca_v_s. Recent studies indicated RIM-BPs (Acuna et al., 2015; Grauel et al., 2016) and Munc13-3 (Kusch et al., 2018) to be involved in organizing Ca_v_ clusters at the AZ and in narrowing the coupling distance. Munc13-3 was identified as a specific developmental mediator of nanodomain coupling at a glutamatergic synapse in cerebellar cortex (Kusch et al., 2018). Interestingly, Munc13-3 protein is expressed strongly in the cerebellar cortex, more weekly in the brainstem and is essentially absent from the hippocampus and cerebral cortex (Augustin et al., 1999). Does this indicate that developmental tightening of the coupling distance is a specificity of glutamatergic synapses in the cerebellum and brainstem? To answer this question, it will be required to quantify coupling distances in a developmental context also at neocortical synapses. Since coupling distances are key parameters of synaptic function, understanding the rules regulating this distance will advance our general understanding of the rules regulating synaptic transmission, which is the basic substrate of information flow in neuronal networks.

## Author Contributions

HS wrote the first draft of the manuscript. GB and HS prepared figures and tables. GB and HS contributed to manuscript revision, read and approved the submitted version.

## Conflict of Interest Statement

The authors declare that the research was conducted in the absence of any commercial or financial relationships that could be construed as a potential conflict of interest.
